# S118. CAN THE POSITIVE AND NEGATIVE SYNDROME SCALE (PANSS) DIFFERENTIATE REFRACTORY FROM NON-REFRACTORY SCHIZOPHRENIA? A FACTOR ANALYTIC INVESTIGATION BASED ON DATA FROM THE PATTERN COHORT STUDY

**DOI:** 10.1093/schbul/sby018.905

**Published:** 2018-04-01

**Authors:** Rosana de Freitas, Bernardo dos Santos, Carlo Altamura, Corrado Bernasconi, Ricardo Corral, Jonathan Evans, Ashok Malla, Marie-Odile Krebs, Anna-Lena Nordstroem, Mathias Zink, Josep Maria Haro, Helio Elkis

**Affiliations:** 1 Universidade de São Paulo; 2 University of Milan, Fondazione IRCCS Ca’ Granda, Ospedale Maggiore Policlinico; 3 F. Hoffmann-La Roche Ltd; 4 Fundación para el Estudio y Tratamiento de las Enfermedades Mentales (FETEM); 5 University of Bristol; 6 McGill University; 7 INSERM, Paris Descartes University, Sainte-Anne Hospital, Service Hospitalo-Universitaire, Paris; 8 Central Institute of Mental Health; 9 Parc Sanitari Sant Joan de Déu, CIBERSAM, Universitat de Barcelona

## Abstract

**Background:**

Treatment Resistant Schizophrenia (TRS) and Non-Treatment Resistant Schizophrenia (NTRS) may represent different biological subtypes of schizophrenia but there are few studies which investigated the distinction between these groups in terms of psychopathology. In the present study, we used both Exploratory (EFA) and Confirmatory (CFA) Factor Analyses to investigate symptom dimensions in TRS in comparison with NTRS using the Positive and Negative Syndrome Scale (PANSS).

**Methods:**

Data from 1429 patients who participated in the PATTERN study a Non- Intervention Prospective Study of Patients with Persistent Symptoms of Schizophrenia) was used. TRS was defined by proxy, based on the use of clozapine (TRS) whereas NTRS used non-clozapine antipsychotics (NTRS). EFA methods included the extraction of principal components and the Varimax rotation. The number of factors was chosen based on the Kaiser criterion. Factors items were considered valid when loadings were greater or equal to 0.5. The fit to the data was evaluated by CFA in comparison with well established PANSS models using fit indexes such as: NNFI (Non-Normed Fit Index), NFI (Normed fit Index), CFI (Comparative Fit Index), RMEA (Root-Mean-Square Error of Approximation). SPSS 23.0 and R version 3.2.2 were used for statistical analyses.

**Results:**

Demographic data showed that, when compared with NTRS, patients with TRS showed an earlier age of onset, a longer duration of illness, higher PANSS positive scores, a higher duration of persistent positive and negative symptoms. There were no differences between groups in terms of the duration of untreated psychosis. The EFA yielded almost the same five-factor structure in both groups namely Negative, Positive, Affective, Disorganized/Cognitive and Excitation factors. CFA showed that both models do not fit completely to the data when compared with well known PANSS factor analytical models.

**Discussion:**

Data from a large cross-national sample of 1429 patients of the Pattern study showed that TRS and NTRS patients have an almost identical factor structure when evaluated by the PANSS. These results are similar to a previous study with a smaller sample which has evaluated the dimensions of the PANSS in patients with refractory schizophrenia.

